# Double trouble: Compound effects of heat and drought stress on carbon assimilation

**DOI:** 10.1093/plphys/kiad647

**Published:** 2023-12-05

**Authors:** Alexandra J Burgess

**Affiliations:** Assistant Features Editor, Plant Physiology, American Society of Plant Biologists; Agriculture and Environmental Sciences, School of Biosciences, University of Nottingham, Sutton Bonington Campus, LE12 5RD Loughborough, UK

Future climate change scenarios predict changes in temperature, water availability, and the frequency and severity of extreme weather events. These climate variables often occur in combination; for example, drought and heat stress generally occur simultaneously. The synergistic impacts of these climate variables often mean that their combined impact on plant physiology is much greater than that of the individual stressors. Therefore, understanding both the individual and compound effect of stressors on carbon assimilation will be vital to both predict and improve crop yield.

Historically, measurement and analysis of photosynthesis has generally occurred under steady-state conditions such as constant high light (Walters 2005). However, the importance of understanding plant performance under natural conditions has highlighted the need to measure dynamic photosynthetic traits. Plant canopies can be structurally complex, resulting in multiple leaf layers, including overlapping foliage. Features of the environment, such as cloud cover or wind, which induces plant movement, results in highly fluctuating light intensities within the canopy (Burgess et al. 2021). The importance of these light dynamics is only just beginning to be recognized. For example, Long et al. (2022) showed that transitions between sun and shade can reduce total carbon assimilation by up to 40%. In addition, genetic approaches to improve the speed of response to light fluctuations produce yield increases of up to 33% under field conditions (De Souza et al. 2022; Kromdijk et al. 2016).

Following an increase in light intensity, photosynthetic rate will increase. The speed of this response, known as the photosynthetic induction rate, is determined by multiple factors, including diffusion of carbon dioxide (CO_2_) into the leaf and cells via stomatal (g_s_) or mesophyll (g_m_) conductance, and through action of the electron transport chain (measured as the electron transport rate [ETR]) and the Calvin Benson Cycle. The factor that limits photosynthetic induction rate is species specific, with variation seen between phylogenetic groups (Tanaka et al. 2019). The main limiting factor for photosynthesis under stressors also differs. For example, under drought stress, a decrease in water potential and simultaneous increase in abscisic acid content reduces g_s_ and g_m_, while fluctuating light can affect stomatal function. In comparison, under heat stress, a reduction in photosynthesis is usually a result of decreased electron transport, reduced functioning of the Calvin Benson Cycle, and increased photorespiration, although heat stress can also affect g_s_ and g_m_.

Within this issue of *Plant Physiology*, Zeng et al. (2023) combined heat and drought stress to determine the mechanisms affecting dynamic photosynthesis of tomato (*Solanum lycopersicum*) under field conditions. Gas exchange measurements under steady-state conditions resulted in an almost 10-fold reduction in maximum assimilation rate under saturating light intensity for plants subject to compound stress (heat and drought) relative to the nonstressed conditions. Similarly, g_s_, g_m_, and ETR exhibited a greater reduction under the compound stress relative to all other treatments.

To determine the impact of single and compound stressors on dynamic photosynthesis, Zeng et al. (2023) measured gas exchange during changes in light intensity. Following 5 min of low light (50 *μ*mol m^−2^ s^−1^), plants were exposed to 1500 *μ*mol m^−2^ s^−1^ for 25 min to quantify photosynthetic induction. Exposure to stressors altered all measured photosynthetic parameters relative to the nonstressed condition (Figure) but to a greater magnitude during compound stress relative to either stress individually. For example, cumulative CO_2_ fixation decreased by up to 50% during either heat or drought stress individually but up to 100% during compound stress. Similar results were also seen for g_m_ and ETR. In comparison, g_s_ exhibited an increase of 70% relative to the control during heat stress but a decrease under both drought and compound stress of 45% and 55%, respectively. Finally, under compound stress, photorespiration acted as a major electron sink, seen as an increase in the contribution of photorespiration to total electron flow for NADPH production (J_o_/J_g_; Figure). Following photosynthetic induction, plants were then exposed to fluctuating light, alternating between 50 and 1500 *μ*mol m^−2^ s^−1^. During fluctuating light, the change in photosynthetic parameters relative to the nonstressed condition exhibited similar patterns to that during photosynthetic induction, namely a greater magnitude of change during compound stressors compared to individual stressors.

**Figure.**
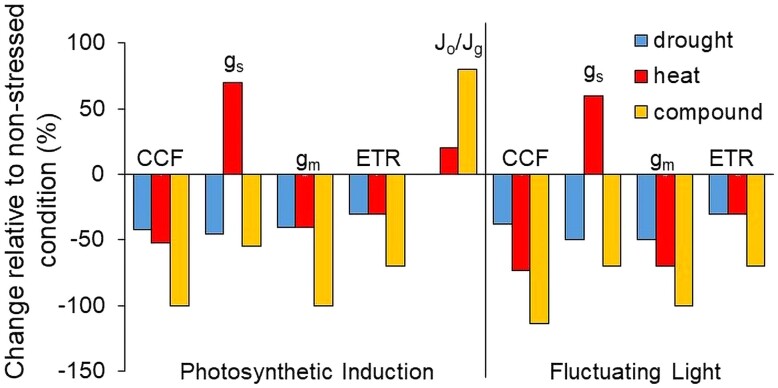
Overview of the impact of drought, heat, and combined drought and heat (compound) stressors, relative to the nonstressed condition, on photosynthetic parameters of tomato (*Solanum lycopersicum*) during dynamic photosynthesis. Parameters cumulative CO_2_ fixation, g_s_, g_m_, ETR, and the contribution of photorespiration to total electron flow for NADPH production (J_o_/J_g_) were measured using an LI6400-XT infra-red gas exchange analyzer following transfer into saturating light (1500 *μ*mol m^−2^ s^−1^) for 25 min (photosynthetic induction: left bars) or during alterations between 50 and 1500 *μ*mol m^−2^ s^−1^ (fluctuating light: right bars).

Together, the results of Zeng et al. (2023) indicate the greater impact of compound stressors on plant physiology than either individual stress. Under combined heat and drought, carbon assimilation during fluctuating light was limited by g_m_. During the high-light phase, g_m_ had a near zero value and thus severely restricted the available substrate for carboxylation reactions. Therefore, future yield improvement strategies should focus on improvements to g_m_ to improve plant performance under combined heat and drought stress of tomato grown under field conditions.

## Data Availability

No new data were generated or analysed in support of this research.

## Dive Curated Terms

The following phenotypic, genotypic, and functional terms are of significance to the work described in this paper:

NADPH CHEBI: CHEBI:16474
